# Efficient attention-based Ghost-ResNet for brain tumor classification in magnetic resonance imaging (MRI)

**DOI:** 10.3389/fnins.2026.1778376

**Published:** 2026-02-23

**Authors:** Nahlah Shatnawi, Khalid M. O. Nahar, Rabia Emhamed Al Mamlook, Ali Saeed Almuflih, Abdullah Mohammed Al Fatais, Salem Alhatamleh, Amal Alishwait, Mohammad Amin

**Affiliations:** 1 Department of Computer Science, Faculty of Information Technology and Computer Sciences, Yarmouk University, Irbid, Jordan; 2 Department of Business Administration, Trine University, Angola, IN, United States; 3 Department of Artificial Intelligence, Center of Advanced Research for Complementary Medicine, University of Zawia, Zawia, Libya; 4 Department of Industrial Engineering, College of Engineering, King Khalid University, Abha, Saudi Arabia; 5 Center for Engineering and Technology Innovations, King Khalid University, Abha, Saudi Arabia

**Keywords:** brain tumor classification, deep learning, efficient channel attention (ECA), Ghost network, medical image, MRI

## Abstract

**Introduction:**

Brain tumor classification from magnetic resonance imaging remains a challenging task in medical image analysis, particularly when high diagnostic performance must be achieved under limited computational resources. Effective models are therefore required to balance classification accuracy with efficiency to support practical clinical deployment.

**Methods:**

This study addresses this challenge by proposing an efficiency-oriented deep learning architecture that integrates Ghost modules into a ResNet-50 backbone and enhances feature learning through Efficient Channel Attention (ECA) blocks. The proposed design aims to improve discriminative capability while reducing feature redundancy and computational overhead.

The model was evaluated on the Bangladesh Brain Cancer MRI Dataset, which contains 6,056 MRI images representing three tumor categories: glioma, meningioma, and pituitary tumors. Preprocessing included contrast normalization using Contrast Limited Adaptive Histogram Equalization (CLAHE). Data augmentation was selectively applied to improve generalization while avoiding excessive artificial amplification of feature representations.

**Results:**

Experimental results demonstrate the effectiveness of the proposed attention-assisted lightweight architecture. The model achieved an overall classification accuracy of 97.85%, while macro-averaged precision, recall (sensitivity), and specificity all exceeded 97.8% (as defined in the Methods section). This corresponds to a 1.65% absolute improvement in accuracy compared with the strongest baseline model, DenseNet121, while maintaining a low false-positive rate. These findings suggest that competitive performance can be achieved without increasing architectural complexity.

**Discussion:**

The results highlight the potential of pursuing efficiency-driven architectural designs as an alternative to increasingly complex deep learning models. In particular, channel-attention-assisted feature generation appears to preserve high diagnostic accuracy while reducing representational and computational overhead, supporting its suitability for resource-constrained medical imaging applications.

## 1 Introduction

Brain tumors remain a persistent challenge in neuro-oncology because of their heterogeneous morphology, variable clinical behavior and substantial effect on patient survival and neurological function. Abnormal cellular proliferation contained by the brain can lead to progressive cognitive decline, motor deficiency and in advanced stages, mortality (Bouhafra and El Bahi, 2025). Epidemiologically, gliomas constitute roughly 22.9% of diagnosed cases however meningiomas account for the largest share at 41.7%; pituitary tumors, even though biologically distinct, present diverse prognostic and therapeutic profiles (Price et al., 2025).

Magnetic Resonance Imaging (MRI) is the modality of choosing for brain tumor evaluation, owing to its superior soft tissue contrast and spatial resolution. Existing clinical protocols integrate multiple opposite sequences T1-weighted, T2-weighted, FLAIR, and contrast-enhanced acquisitions to support tumor description and treatment planning (Kanal et al., 2025). Regardless of these advances, MRI interpretation continues largely manual, time-intensive and dependent on specialist expertise which limits diagnostic scalability, particularly in resource-constrained healthcare settings (Aiya et al., 2025).

A number of uncertain tasks are present within the process of computerized brain tumor classification based on MRI images. The presence within the brain tumor categories of high intra-, as well as inter-class variations regarding the attributes of size, type, consistency, and position, improves the difficulty level in this task (Albalawi et al., 2024). These issues are alleviated by the presence of high variations within the process of data acquisition, as well as the scanner-dependent factors, which are known to adversely affect the process of generalization (Kanal et al., 2025). Apart from this, the occurrence of class imbalance and restricted expert annotation regarding the distinctive types of brain tumors is a constraint within real-world clinical settings, restricting the consistency levels of the models (Disci et al., 2025). From the clinical point of view, efficiency is equally important, as systems that consume more resources are not easily computable within clinical settings (Babu Vimala et al., 2023).

Deep learning has restructured medical image analysis with Convolutional Neural Networks (CNNs), developing as the dominant structure for MRI-based tumor classification (Albalawi et al., 2024). Transfer learning has been shown to centrally contribute to this progress, assisting pretrained constructions such as VGG, ResNet, and DenseNet to be adapted to medical imaging commissions despite limited labeled data (Babu Vimala et al., 2023; Disci et al., 2025). More recent developments incorporate attention mechanisms and hybrid CNN–Transformer designs to improve feature discrimination and contextual modeling. In parallel, lightweight architectures and modules focusing on efficiency have emerged as interesting approaches to minimize compute overheads without compromising the accuracy of diagnosis, a consideration that has increasing significance in practical clinical use scenarios (Han et al., 2020; Ferdous et al., 2025).

Despite the remarkable progress achieved by deep learning techniques in brain tumor classification using MRI, most state-of-the-art models still rely on deep and computationally expensive architectures. Such approaches often prioritize accuracy improvements at the cost of increased parameter size, memory consumption, and inference time, which limits their applicability in real-world clinical environments, particularly in resource-constrained settings. Moreover, several existing studies employ aggressive data augmentation and feature expansion strategies that may artificially inflate performance while increasing computational overhead. These gaps underscore the need for lightweight yet highly discriminative models that can strike a balance between classification accuracy and computational efficiency. Motivated by these limitations, this study aims to design an efficiency-oriented architecture that integrates Ghost modules with a ResNet backbone and enhances feature discrimination through Efficient Channel Attention (ECA), thereby achieving high diagnostic accuracy without sacrificing computational feasibility.

Based on the identified research gaps, this study seeks to address the following research questions: (1) Can the integration of Ghost modules into a ResNet-based architecture significantly reduce computational complexity while preserving or improving classification performance for brain tumor MRI images? (2) To what extent does the incorporation of Efficient Channel Attention (ECA) improve feature representation and tumor discrimination in a lightweight network? (3) How does the proposed attention-assisted Ghost-ResNet model compare with existing lightweight and standard deep learning architectures in terms of accuracy, precision, recall, specificity, and false positive rate? Answering these questions will help determine whether an efficiency-driven architectural design can challenge the prevailing assumption that higher accuracy necessarily requires deeper and more computationally intensive models.

This paper deals with the existing difference between performance-based consideration and usability in the clinical use scenario of brain tumor image classification using automated systems, with a focus on maximizing diagnosis accuracy along with the aspects of computational efficiency, robustness to variability in imaging acquisition, and practical clinical usability. The contributions of this paper are given below:

1. The study introduces an efficient attention-based deep learning framework that integrates a Ghost-enhanced ResNet backbone with channel-wise attention to improve discriminative feature learning while reducing computational redundancy.
2. A targeted MRI preprocessing and reinforcement plan is utilized to improve image quality, stabilize training, and expand generalization across heterogeneous clinical data.
3. Wide-ranging experimental evaluation validates that the proposed method reliably outperforms established transfer-learning baselines, realizing superior accuracy, sensitivity and specificity while maintaining suitability for real-world clinical implementation.

The remainder of this paper is organized as follows. Section 2 reviews related work in deep learning-based brain tumor classification. Section 3 details the dataset, preprocessing strategy, and proposed architecture. Section 4 illustrates investigational results and relative analyses. Section 5 reflects the results in a broader clinical and technical setting and section 6 concludes with final notes and directions for future research.

## 2 Related work

Deep learning has been identified as the most preferred methodology found to date in classifying brain tumors from magnetic resonance images, because of its capability to learn hierarchical representations without resorting to engineered features (Litjens et al., 2017; Shen et al., 2017). Traditional computer-assisted diagnosis solutions proposed and designed around texture features, wavelet transforms, or statistical feature extraction lacked robustness when exposed to variations existing within the image modality, as well as those within the tumors (Zacharaki et al., 2009). Convolutional neural networks (CNNs) have since displaced these approaches by enabling end-to-end optimization directly on imaging data. Despite rapid methodological progress, a central limitation persists across both artificial intelligence and clinical domains: the prevailing dependence on increasingly deep and computationally expensive architectures to achieve marginal performance gains. Table 1 displays a comparison with previous studies on brain tumor Classification.

**TABLE 1:** Comparison of previous studies of brain tumor classification.

References	Dataset	Methodology	Reported accuracy	Key limitations
Shahin (2025)	Figshare (7,023 images, 4 classes)	Fine-tuned ResNet-34 with Ranger optimizer	99.66%	Single-model evaluation; limited dataset diversity
Aiya et al. (2025)	Figshare (7,023 images, 4 classes)	VGG16 with Attention mechanisms and Grad-CAM	99.00%	Complex preprocessing; explainability overhead
Babu Vimala et al. (2023)	Multiple datasets (3 classes)	EfficientNet with transfer learning and fine-tuning	99.06%	Dataset-dependent generalization
Wong et al. (2025)	17,136 augmented MRI images (4 classes)	Pretrained VGG16 with extensive data augmentation	99.24%	High computational and augmentation cost
Disci et al. (2025)	Figshare (7,023 images, 4 classes)	Comparative study of multiple CNN architectures	98.73% (Xception)	Sensitivity to class imbalance
Zahoor et al. (2024)	Kaggle, Br35H, Figshare	Res-BRNet (Residual + Regional CNN)	98.22%	Requires broader external validation
Rastogi et al. (2025)	Kaggle brain tumor dataset	Fine-tuned transfer learning (InceptionResNetV2, VGG19, Xception, MobileNetV2)	96.11%	Performance variability across models
Ahsan et al. (2025)	Brain Tumor Figshare dataset	YOLOv5 detection combined with 2D U-Net segmentation	89.5% mAP/88.1% DSC	Detection–segmentation dependency
Vamsidhar et al. (2025)	Multiple MRI datasets	Hybrid ResNet101 + Xception with LIME/ViT + RF	99.67%	Ensemble complexity; high inference cost
Iftikhar et al. (2025)	Figshare (7,023 images, 4 classes)	XAI + CNN	99% accuracy on seen data and 95% on unseen data	Added complexity for deployment
Khandaker et al. (2024)	Multi-class Brain MRI datasets	Nine-model ensemble with XAI techniques	99.83% (DenseNet169)	Very high computational overhead

A substantial body of work reports high classification accuracy through architectural modification of established CNN backbones. Residual learning–based models, such as modified ResNet-34 architectures with task-specific classification heads, have achieved accuracy levels as high as 99.66% on publicly available MRI datasets, demonstrating the effectiveness of skip connections for stabilizing deep feature learning (Shahin, 2025). Attention-enhanced VGG-based hybrid frameworks further improve discrimination by reweighting salient feature channels and spatial regions, with reported accuracies approaching 99% on Kaggle and BRaTS datasets (Aiya et al., 2025). However, these architectures often rely on extensive data augmentation and large parameter counts, limiting computational efficiency and increasing sensitivity to preprocessing choices.

Efficiency-driven transfer learning has been discovered throughout compound-scaled architecture. EfficientNet-based models applying coordinated scaling of network depth, width, and input resolution concession strong performance for multi-class brain tumor classification, with reported test accuracy exceeding 99% (Babu Vimala et al., 2023). While such designs improve parameter utilization, they still incur nontrivial inference costs and memory footprints, posing challenges for deployment in real-time or resource-constrained clinical environments.

Comparative benchmarking analyses that base performance have a strong dependency in relation to the composition of data, preprocessing steps, and metrics used in model assessment. Large-scale comparative analyses among pretrained models such as Xception, MobileNetV2, InceptionV3, ResNet50, and DenseNet models resolve several top-performing models depending upon various experimental settings (Disci et al., 2025; Wong et al., 2025). Such results point out the lack of architectural robustness and raise issues related to the generalizability paradigm.

Contemporary trends in research involve the use of hybrid and region-aware approaches. The combination of residual learning and region-based convolutional layer activities improves spatial and boundary awareness, hence increasing the capability for discrimination in diverse tumor morphologies (Zahoor et al., 2024). Other approaches involve the combination of the detection and segmentation pipeline, including the use of YOLOv5 with the two-dimensional U-Net architectures, resulting in enhanced Dice coefficient and localization accuracy compared with the classical Mask R-CNN model. This function increases pipeline complexity and reduces model reproducibility (Ahsan et al., 2025; Rastogi et al., 2025).

Recent studies have demonstrated the effectiveness of convolutional neural networks (CNNs) for automated medical image classification (Meena et al., 2023). In the context of brain tumor detection, an improved CNN-based model was proposed to address binary classification of MRI scans by distinguishing tumor and non-tumor cases. The study leveraged data augmentation strategies to improve classification accuracy and reduce training time, reporting strong performance across multiple evaluation metrics such as accuracy, precision, recall, and F1-score. While this work highlights the benefit of augmentation and efficient CNN design, it focuses on binary classification and does not explicitly address computational redundancy or efficiency constraints in multi-class tumor classification scenarios.

Beyond brain tumor imaging, deep learning techniques have also been explored for abnormality detection in other radiological domains. For example, a study on musculoskeletal radiographs employed DenseNet and VGG architectures to identify abnormalities using the large-scale MURA dataset (Choudhary and Meena, 2021). The authors compared their models against those from the Stanford ML Group MURA Competition using the Cohen Kappa statistic and demonstrated that deep CNNs can achieve performance comparable to or exceeding state-of-the-art approaches in several study types. However, such architectures remain computationally intensive and are not optimized for lightweight or efficiency-oriented deployment.

In contrast to these existing works, the present study focuses on balancing diagnostic accuracy with computational efficiency by integrating Ghost modules and Efficient Channel Attention within a residual learning framework. This design explicitly targets the reduction of redundant feature generation while preserving discriminative power, enabling effective multi-class brain tumor classification under constrained computational budgets.

Interpretability has increasingly attracted attention, thanks to the need in medical applications for interpretability and trust. Parallel CNN ensembles and post-hoc explanation methods, such as LIME and explainable AI methods (XAI), have been used to identify decision-relevant areas (Vamsidhar et al., 2025). It should, however, be noted that interpretability in these experiments has generally been considered a posteriori or built into specific CNN architectures. Interpretable CNN architectures have shown moderate improvements through extensive regularization and optimization, reaching a maximum of 97.1% accuracy with models based on DenseNet (Iftikhar et al., 2025). Large-scale evaluations across nine pretrained CNNs continue to favor deep, parameter-intensive networks when accuracy alone is optimized (Khandaker et al., 2024).

The literature has already proven that near-saturate accuracy can be realized; however, this accuracy usually costs dearly in terms of architectures that are computationally expensive, transparently complicated, and ill-aligned with real-world deployment needs. The open research question fundamentally involves creating architectures that are naturally attentive, inherently bounded with regards to representational redundancy, and faithful to medical diagnosing only through meaningful architectural efficiencies with direct implications for real-world medical innovation and not purely through accuracy gains. The need exists to move away from accuracy-specific scaling and toward relevant architectural efficiencies.

## 3 Materials and methods

**FIGURE 1:**
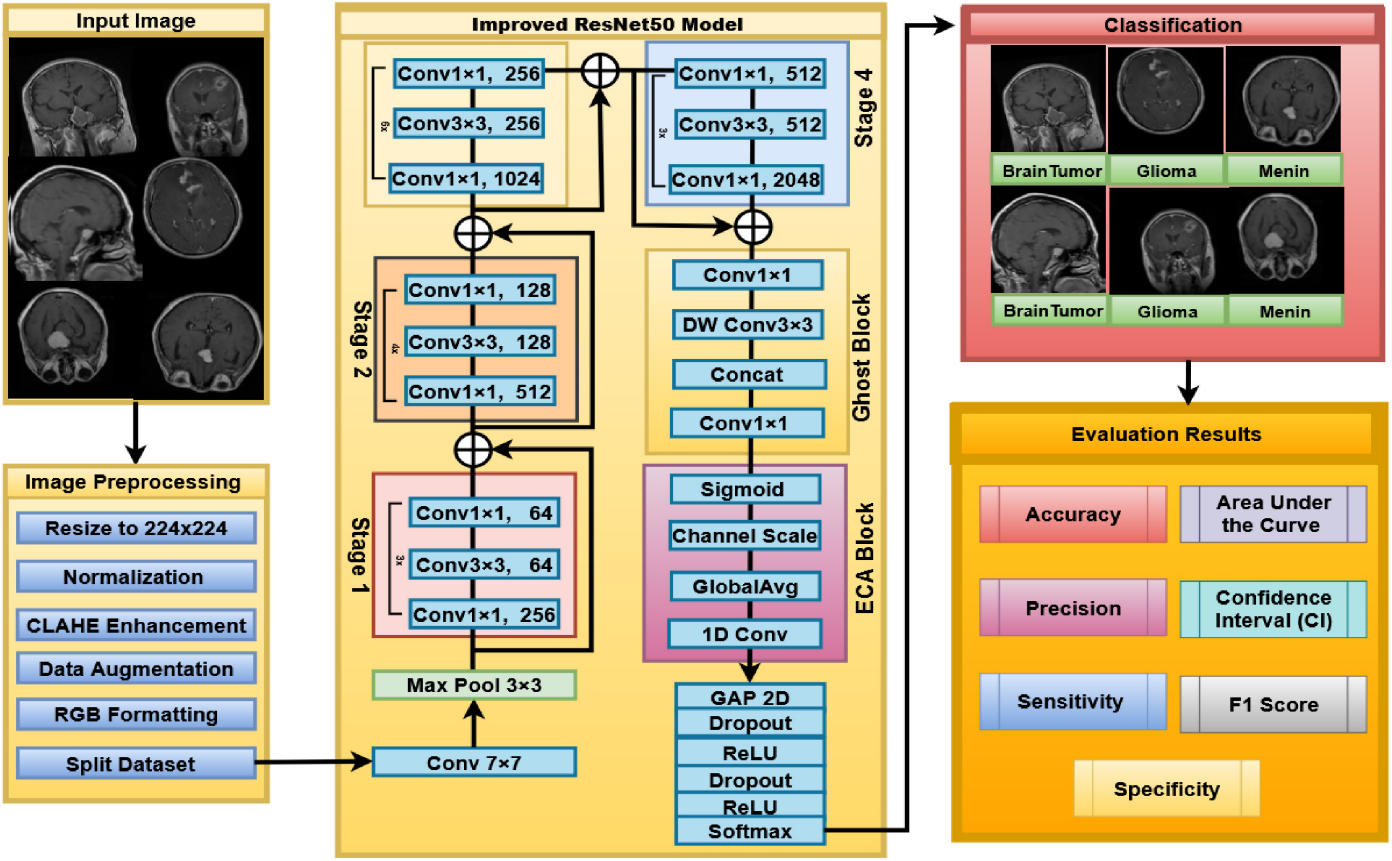
Overview of the proposed improved ResNet50 architecture for brain tumor classification from MRI images.

The proposed model has a series of strongly integrated steps in order to efficiently classify the brain tumor from MRI scans. Step 1 involves the normalization of the input image by resizing into 224 × 224 resolution images, intensity normalization, contrast enhancement using CLAHE, along with specific data augmentation strategies. This is aimed at reducing the variability of contrast induced during the acquisition procedure while retaining the anatomical structures. Step 2 involves the use of the improved ResNet50 architecture to learn the spatial and semantic features from the input image. It uses residual connections to promote stable optimization. Step 3 involves the incorporation of Ghost blocks into the model. They work on the principle of using lightweight depth-wise operations to discard the redundancy of features. Step 4 involves the use of ECA (Efficient Channel Attention), a strategy that enhances the model features using the entire context. It boosts the desired features. Step 5 involves the use of a classifier that involves a lobal average pooling followed by a regularized classification head (e.g., dropout/weight decay). Its performance is measured using accuracy, precision, sensitivity, specificity, F1-score. As illustrated in Figure 1 the model pipeline begins with MRI inputs that are processed through a deep convolutional backbone to extract high-level spatial and semantic feature representations.

### 3.1 Dataset and preprocessing

#### 3.1.1 Dataset collection

**FIGURE 2:**
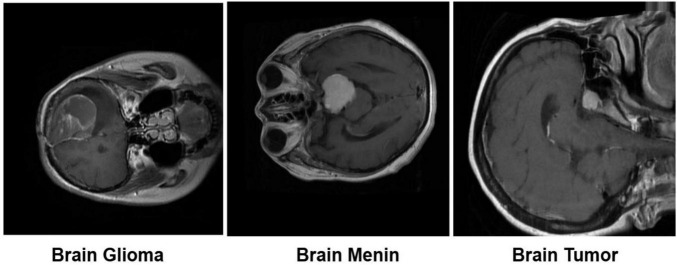
Dataset representation.

The experimental evaluation was directed using the Bangladesh Brain Cancer MRI Dataset which contains 6,056 magnetic resonance images distributed across three related categories: glioma (2,004 images), meningioma (2,004 images) and pituitary related brain tumors (2,048 images) (Rahman, 2024). The dataset was collected through collaborations with multiple hospitals across Bangladesh and met in consultation with medical specialists to guarantee diagnostic validity and clinical relevance. All images were uniformly resized to a spatial resolution of 224 × 224 pixels providing a consistent input format while preserving anatomically meaningful detail. Remaining to its multi-institutional origin and balanced class composition, the dataset gains a representative range of tumor attendances and imaging conditions met in routine clinical practice. These attributes make it well suited for the development and evaluation of computerized diagnostic models in brain tumor imaging. An 80/10/10 split ratio was applied at the patient level, ensuring that no subject overlap exists across subsets. This strategy prevents correlated samples from the same patient appearing in multiple splits and avoids artificially inflated performance estimates (Yagis et al., 2021). Typical MRI samples from each tumor category are explained in Figure 2.

#### 3.1.2 Preprocessing image

Image preprocessing is a very basic step in the process of stabilizing the pipeline for deep learning, and this is especially true when it comes to using MRI images in the medical or healthcare sector, wherein MRI images are generally prone to noise, contrast, and artifacts based on the acquisition process. Even though there are certain sources of uncertainty, the brain MRI images have been exposed to a series of correctly chosen techniques based on the need for spatial resolution, local contrast enhancement, noise reduction, and clear demarcation around the edges of the tumor. All the images had to undergo resizing based on a unified spatial resolution of *I* ∈ ℝ^224×224×3^ so that there would be complete congruence with the ResNet-50 backbone network and no inconsistencies with tensor dimensions while undergoing forward and backward passes. The pixel intensity values, which lie in the range [0, 255], have been converted to the range [0, 1]. However, this process has the effect of stabilizing the gradient magnitudes and, therefore, increases the speed of convergence and prevents possible instability of the optimization algorithm due to gradient computations (Li et al., 2021). For this reason, because of the intrinsic nature of low-contrast images of the kind produced by MRI, Contrast-Limited Adaptive Histogram Equalization (CLAHE) had to be carried out (Zuiderveld, 1994). Images were first converted from RGB color space to the LAB representation; subsequently, CLAHE was performed only on the luminance channel, which was then combined with chromatic information and transformed back to RGB space (Mishra, 2021). This procedure significantly enhances the delineation of tumor margins and internal texture while maintaining consistency in global intensity.


$$I_{L\,A\,B}=f_{R\,G\,B\to L\,A\,B}\,\left(I\right)$$ (1)

$$C\,D\,F\,\left(k\right)=\overset{k}{\underset{i=0}{\sum }}\frac{h\,\left(i\right)}{N}$$ (2)

In this case, the local intensity histogram is expressed by *h*(*i*), with *N* being the total number of pixels for each contextual image portion. The resulting display is utilized for calculating the adjusted luminance values; afterwards, the enhanced light image is combined with the chrominance channels to revert to the RGB color space. The approach expands the definition of the tumor boundaries and reveals the tumor heterogeneity, while the adaptive clipping limits the contrast enhancement and prevents overemphasis. Before feature extraction, Gaussian filtering was employed to attenuate high-frequency noise introduced during image acquisition and to suppress unrelated background fluctuations. Smoothing of images was done by convolution with a Gaussian kernel. The standard deviation parameter σ controls the amount of smoothing. This operation effectively reduces random noise while retaining critical anatomical structures that are important for reliable downstream feature learning.


$$L^{{}^{\prime }}\,\left(k\right)=\left(L_{\text{max}}-1\right)\cdot C\,D\,F\,\left(k\right)$$ (3)

$$G\,\left(x,y\right)=\frac{1}{2\,\pi \,\sigma^{2}}\,\text{exp}\,\left(-\frac{x^{2}+y^{2}}{2\,\sigma^{2}}\right)$$ (4)

$$I_{\text{blur}}\,\left(i,j\right)=\underset{u,v}{\sum }G\,\left(u,v\right)\,I\,\left(i-u,j-v\right)$$ (5)

Following noise attenuation, tumor boundary definition was selectively intensified using unsharp masking, a technique designed to accentuate high-frequency structural details without distorting underlying anatomy. In this stage, a smoothed calculation of the original image (*I*_*blur*_) is first generated, after which edge information is secluded by withdrawing the obscured component from the original signal. The detail layer is then reintegrated to produce a sharpened image, yielding clearer lesion contours and improved spatial discrimination while preserving tissue integrity.


$$I_{\text{detail}}=I-I_{\text{blur}}$$ (6)

$$I_{\text{sharp}}=I+k\cdot I_{\text{detail}}$$ (7)

In this case, *K* represents a scalar value responsible for controlling the strength of boundary enhancement, which has a direct impact on boundary intensity and, consequently, image spatial discrimination. However, this enhancement procedure alone leads to only a moderate and somewhat blurry definition of boundaries in favor of image spatial discrimination and amplifies convergent structural details by emphasizing second-order intensity variations with Laplacian operators without compromising image spatial discrimination. To enhance image coherence and reduce irregularities in intensity distributions inside tumors, a morphological closing procedure was performed to reduce irregularities inside tumors, followed by second-order intensity variation enhancement with Laplacian operators to further emphasize structural details with a direct impact on image spatial discrimination. A morphological closing, performed to enhance image coherence and reduce irregularities, represents a combination of dilations and erosions with a given structuring element *K*, which, in this case, is elliptical, providing a direct impact on fill-in processes of small cavities inside tumors and image spatial discrimination, as follows:


I⋅K=(I⊕K)⊖K (8)

$$\nabla \alpha^{2}\,I=\frac{\alpha^{2}\,I}{\alpha \,x^{2}}+\frac{\alpha^{2}\,I}{\alpha \,y^{2}}$$ (9)

$$I_{\text{enhanced}}=I+\alpha \cdot \nabla^{2}I$$ (10)

The value of αdetermines the degree to which edge information is highlighted, making it possible to adjust the importance of the structure without sharp edges being created artificially. The step-by-step process of improvement, noise removal, edge enhancement, and morphological optimization helps to achieve images with prominently improved quality concerning the clarity and definition of the tumor region. The whole preprocessing step makes it possible to increase the definition of the boundaries and the visibility of the tissue inside, achieving a representation that is suitable only for the extraction of deep features.

### 3.2 Model building

ResNet-50, despite its great deep feature extraction capability, has a high computational cost, feature redundancy, and no clear attention mechanism as its drawbacks. These drawbacks lead to a lesser extent of efficiency and less capability to bring out the tumor-relevant features in brain MRI images. The Ghost Module is integrated to tackle these issues by removing redundant feature maps and cutting down the computational load via a cheap feature generation. Simultaneously, the Efficient Channel Attention (ECA) technique is utilized to adaptively channel-wise features and boost the tumor’s discriminative representations. The integrated structure allows the proposed architecture to deliver heightened efficiency, precision, and selectivity in features.

ResNet-50 is a deep convolutional architecture comprising fifty layers and is founded on the principle of residual learning, originally introduced to mitigate degradation and vanishing-gradient phenomena in very deep networks (Wen et al., 2020). Rather than learning a direct mapping *H*(*x*), each residual block is formulated to learn a residual function *F*(*x*), such that the block output is defined as


y=F⁢(x)+x (11)

where *x*denotes the input feature map and *F*(*x*) represents the transformation learned by the stacked convolutional layers. The identity shortcut connection enables gradients to propagate directly through the network during backpropagation, stabilizing optimization and facilitating the effective training of deep architectures (Liu et al., 2023).

Each bottleneck residual block in ResNet-50 consists of a sequence of three convolutional layers arranged in a 1×1, 3×3, and 1×1 configuration. The initial 1×1 convolution performs channel dimensionality reduction, the 3×3convolution extracts spatial features, and the final 1×1convolution restores the original channel depth. Given an input tensor X ∈ ℝ^*H*×*W*×*C*^, the bottleneck transformation is expressed as


$$F\,\left(\text{X}\right)={\text{W}}_{3}*\sigma \,\left({\text{W}}_{2}*\sigma \,\left({\text{W}}_{1}*\text{X}\right)\right),$$ (12)

where W_1_,W_2_,W_3_ denote convolutional kernels, *represents convolution, and σ(⋅) is the ReLU activation. The block output is obtained via residual summation:


Y=F⁢(X)+X. (13)

Batch normalization is employed after every single convolutional layer to normalize feature distributions and accelerate convergence. Concluded succeeding stages, ResNet-50 gradually encodes visual information, starting with low-level edges and passion gradients, advancing to mid-level texture and structural patterns and culminating in high-level semantic illustrations associated with tumor morphology and spatial extent. For an input MRI image I (Yadav et al., 2024), the backbone produces a deep feature tensor


F=ResNet-50⁢(I), (14)

where F ∈ ℝ^*H*′×*W*′×*C*′^ serves as the foundational representation for subsequent feature refinement and attention mechanisms.

**FIGURE 3:**
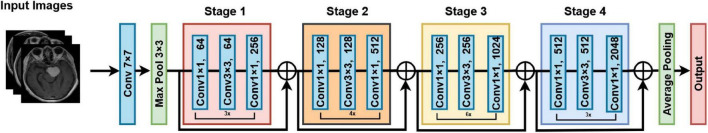
Detailed schematic of the ResNet-50 backbone, illustrating progressive feature extraction from MRI input to output prediction.

As illustrated in Figure 3 the network begins with a 7×7 convolution followed by 3×3 max pooling to extract initial features. It then traverses four residual stages (Stages 1–4), each composed of multiple bottleneck blocks with skip connections to preserve information flow and suppress gradient attenuation. Global average pooling precedes the output layer, which generates the final classification prediction.

#### 3.2.2 Ghost module

The Ghost Module is introduced to specifically handle the high computational complexity and feature redundancy caused by traditional convolutional layers (Chen et al., 2022). The key idea behind the Ghost Module is that most generated feature maps by traditional convolutional layers are strongly correlated and, hence, unnecessary to calculate through the convolutional operation (Han et al., 2020). Contrary to traditional convolutional layers, where the entire result is computed, the Ghost Module proposes the generation process to consist of two tasks: the identification of intrinsic feature maps and the production of the rest by low-cost transformations.

Formally, given an input feature tensor, a standard convolution produces an output **Y** ∈ ℝ^*H*×*W*×*C*^ via the operation


$$Y=W^{*}\,X$$ (15)

where W denotes the convolutional kernel and *represents convolution. This operation becomes increasingly expensive as the number of output channels *C*_*out*_ grows. In contrast, the Ghost Module first generates a reduced set of intrinsic features F_*p*_ using a lightweight convolutional kernel W_*p*_, producing *C*_*int*_ channels, where *C*_int_≪*C*_out_:


$$F_{p}=W_{p}*X,$$ (16)

The remaining feature maps, referred to as ghost features, are obtained by applying inexpensive linear transformations Φ(⋅), typically implemented as depthwise convolutions:


$$F_{g}=\Phi \,\left(F_{p}\right),$$ (17)

The intrinsic and ghost features are then concatenated,


$$F_{\text{ghost}}=\left[F_{p},F_{g}\right].$$ (18)

and subsequently fused using a 1=1 convolution:


$$Y=W_{1\times 1}*F_{\text{ghost}}.$$ (19)

It significantly alleviates the floating-point calculations and the number of parameters compared with the conventional convolutions while maintaining the representational ability (Chen et al., 2022). In the brain MRI classification problem, the Ghost Module helps to efficiently suppress the redundant feature propagation introduced by the ResNet-50 backbone network, allowing the model to pay more attention to the tumor-related features.

#### 3.2.3 Efficient channel attention

To further improve features, the suggested architecture incorporates the Efficient Channel Attention (ECA) module (Wang et al., 2020). Different conventional attention mechanisms that depend on reduction and completely associated transformations, ECA approves a lightweight, channel-wise strategy that preserves the original channel dimensionality while capturing inter-channel dependencies at minimal computational cost. This design choosing is mostly advantageous in medical imaging where subtle intensity and texture variations carry diagnostic significance.

Given an input feature tensor X ∈ ℝ^*H*×*W*×*C*^, ECA first applies Global Average Pooling (GAP) to condense spatial information into a compact channel descriptor (Bakr et al., 2022). For each channel *c*, the descriptor is computed as


$$z_{c}=\frac{1}{H\,W}\overset{H}{\underset{i=1}{\sum }}\Sigma_{j=1}^{W}x_{i,j,c},c=1,2,\ldots ..,C$$ (20)

forming the channel vector


$$\text{z}=\left[z_{1},z_{2},,z_{C}\right]\in {\mathbb{R}}^{C}.$$ (21)

Rather than employing fully connected layers, ECA models local cross-channel interactions using a one-dimensional convolution with an adaptively determined kernel size *k*. The kernel size is defined as


$$k={\left|\left({\text{log}}_{2}\,C\right)\,\gamma +b\right|}_{\text{odd}},$$ (22)

where γand *b*control the receptive field and |⋅|_odd_ enforces an odd kernel size to maintain symmetric convolution. The resulting attention weights are passed through a sigmoid activation and applied to rescale the original feature tensor:


$$x_{i,j,c}^{{}^{\prime }}=a_{c}\cdot x_{i,j,c},$$ (23)

where *a_c_*denotes the learned importance of the *c*-th channel.

It is observed in adaptive reweighting, where the process favors the enhancement of tumor-related channels and suppresses the contributions of less informative background channels. But more importantly, ECA does all this with a computation cost practically equivalent to a squeeze-and-excitation (SE) block, when precision is a crucial concern in a resolution-heavy medical image classification problem.

#### 3.2.4 Integrated model architecture and training framework

In particular, the proposed architecture is built by the conscious incorporation of three complementary components: a backbone of ResNet-50, a Ghost feature generation module, and a mechanism of Efficient Channel Attention. Such a design addresses the main limitations that are conventionally associated with standard deep convolutional networks in the context of medical image classification, such as high computational cost, feature redundancy, and lack of explicit attention modeling. ResNet-50 was chosen as the important feature extractor exactly for its recognized capability to realize hierarchical spatial and semantic representations. However, direct utilization of the backbone directly provides dense feature maps with significant redundancy and low interpretability.

For the mitigation of these issues, the output feature maps from ResNet-50 are transformed by the Ghost Module. This module reformulates the convolution process by forming a small set of inherent feature maps and extracting the others from them through low-cost linear transformations. This practice significantly reduces the generation of unnecessary features while retaining expressiveness, resulting in a significantly reduced computational cost without impairing discrimination. After Ghost-based Refining, channel-wise feature recalibration was carried out through Efficient Channel Attention. ECA attentioned that ResNet-50 inherently flattened all channels equally; thus, data-driven channel weighing was encouraged in ECA to emphasize selected channels that are dwelt in tumorous conditions by down-weighting channels that are dwelt in the background scenes.

The refined representations obtained then undergo global average pooling and then pass through a fully connected classification head, forming an end-to-end trainable system. Optimization of the models has been conducted using supervised mini-batch gradient descent with Adam optimization to ensure convergent performance. Dropout regularization has also been adopted to handle overfitting issues. Combining all these modules together, this comprehensive system addresses the primary weakness of ResNet-50, which consists of computational inefficiency, feature redundancy, and poor attention sensitivity, with a fair balance between accuracy, robustness, and computational efficiency.

In the proposed architecture, feature embeddings are obtained from the high-level convolutional feature maps produced after the Ghost-ResNet backbone enhanced with Efficient Channel Attention blocks. These embeddings represent compact, high-dimensional representations of MRI scans, capturing both spatial and channel-wise discriminative information. Global Average Pooling (GAP) is applied to transform the feature maps into fixed-length embedding vectors, which are then fed into the final classification layers for multi-class tumor prediction.

### 3.3 Implementation and training details

The proposed Efficient Attention-Based Ghost-ResNet model was implemented using the PyTorch deep learning framework and trained under unified experimental settings to ensure reproducibility and fair comparison with baseline models. Training was conducted for 10 epochs using the Adam optimizer with an initial learning rate of 1 × 10^–4^ and a batch size of 32, while categorical cross-entropy was employed as the loss function for multi-class classification. To reduce overfitting, a weight decay of 1 × 10^–5^ and a dropout rate of 0.5 in the fully connected layers were applied. The network was initialized with ImageNet-pretrained weights, where early convolutional layers were initially frozen to preserve generic feature representations, followed by gradual unfreezing of higher-level layers for fine-tuning using a reduced learning rate. A learning rate scheduler was utilized to decrease the learning rate upon stagnation of the validation loss. All experiments were performed using a fixed random seed to ensure consistent results and were executed on a workstation equipped with an NVIDIA GPU and 32 GB of RAM.

### 3.4 Evaluation metrics and statistical analysis

The performance of the models has been evaluated using a variety of statistics to ensure that the models are evaluated effectively and fairly (Hossin and Sulaiman, 2015). Finally, the proposed model has been validated on a test dataset containing MRI scans that are not used during training to prevent any leakage of information. Let TP, TN, FP, and FN represent the true positives, true negatives, false positives, and false negatives, respectively.

Overall classification accuracy was adopted as the primary metric, as it reflects the proportion of correctly classified instances across all tumor categories:


$$A\,c\,c\,u\,r\,a\,c\,y=\frac{T\,P+T\,N}{T\,P+T\,N+F\,P+F\,N}$$ (24)

While accuracy provides a global measure of correctness, it may obscure class-specific behavior, particularly in multi-class or imbalanced settings. To address this limitation, precision and sensitivity were additionally reported to capture predictive reliability and detection capability. Precision quantifies the proportion of correctly identified positive cases among all predicted positives:


$$P\,r\,e\,c\,i\,s\,i\,o\,n=\frac{T\,P}{T\,P+F\,P}$$ (25)

Sensitivity, also referred to as recall, measures the model’s ability to correctly identify true positive cases:


$$\text{Sensitivity}=\frac{T\,P}{T\,P+F\,N}$$ (26)

Specificity was included to evaluate correct identification of negative cases:


$$\text{Specificity}=\frac{T\,N}{T\,N+F\,P}$$ (27)

Because precision and sensitivity capture complementary aspects of performance, the F1-score was computed as their harmonic means to provide a balanced summary metric:


$$F\,1=\frac{2\cdot P\,r\,e\,c\,i\,s\,i\,o\,n\cdot R\,e\,c\,a\,l\,l}{P\,r\,e\,c\,i\,s\,i\,o\,n+R\,e\,c\,a\,l\,l}$$ (28)

## 4 Results

The proposed model was fine-tuned using transfer learning. The initial layers of the backbone network were initialized with ImageNet-pretrained weights and frozen during early training stages to preserve low-level feature representations. Subsequently, deeper layers were gradually unfrozen and fine-tuned using a lower learning rate to adapt the model to MRI-specific features. Adam optimizer was employed with a learning rate scheduling strategy to ensure stable convergence. This progressive fine-tuning approach significantly improved convergence speed and classification performance.

The proposed construction was estimated on the Bangladesh Brain Cancer MRI Dataset which involves of 6,056 images distributed across three tumor categories: glioma (2,004 images), meningioma (2,004 images) and pituitary tumors (2,048 images). The dataset was subdivided into training, validation and testing subsets using an 80/10/10 split. Model optimization was accomplished over 10 epochs, utilizing the Adam optimizer with a learning rate of 1×10^−4^. Performance was assessed using accuracy, precision, sensitivity (recall), specificity, and F1-score to ensure a comprehensive and meaningful evaluation of classification behavior.

### 4.1 Comparative analysis against transfer-learning baselines

**FIGURE 4:**
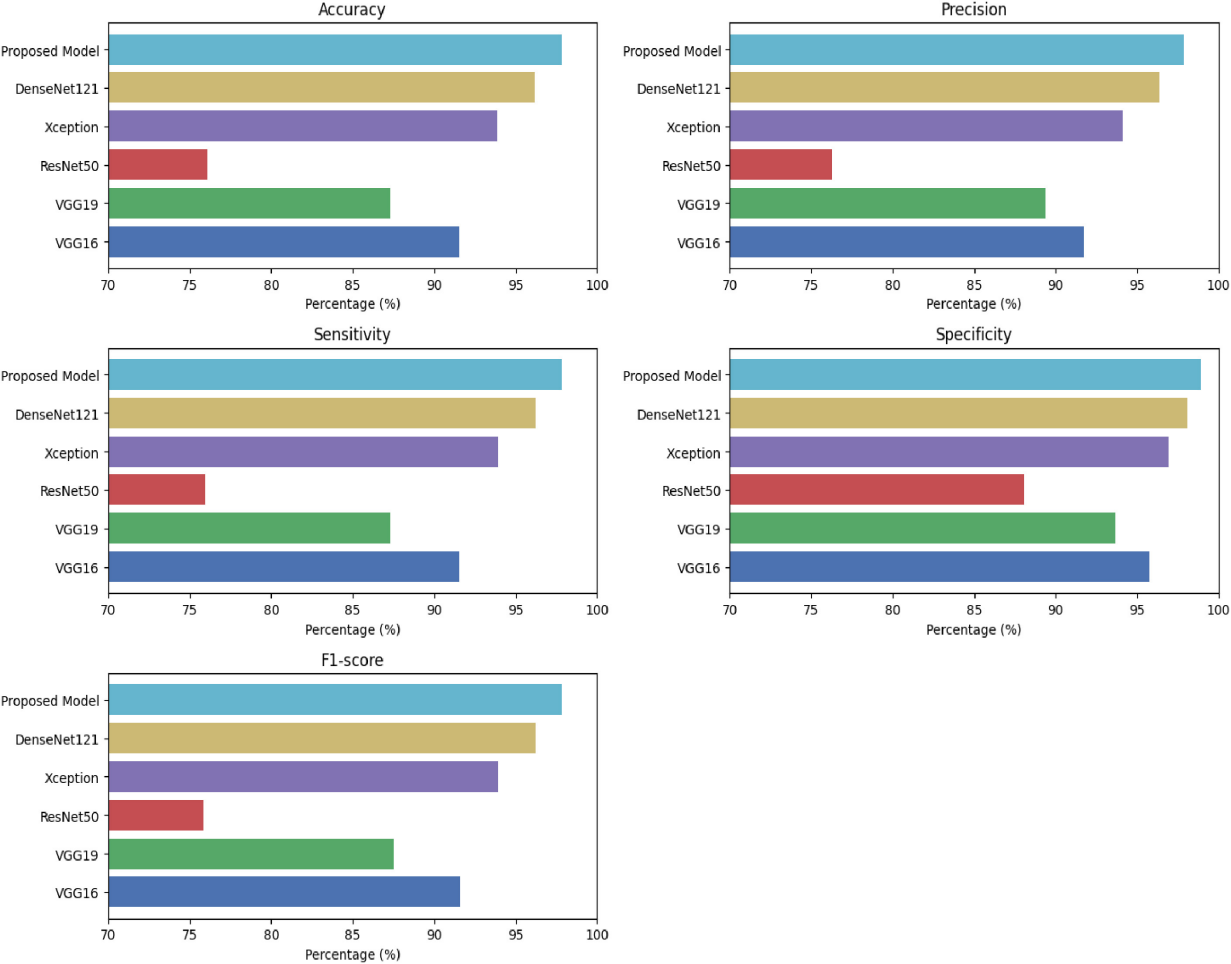
Comparative assessment of classification performance across baseline and proposed models using clinically relevant evaluation metrics.

The comparative results summarized in Table 2 and visualized in Figure 4 can be interpreted through the lens of Efficiency Accuracy Trade-off Theory, which posits that predictive performance in deep learning does not increase linearly with network depth or parameter count once representational saturation is reached. VGG16 occupies a middling position realizing 91.58% accuracy with precision and sensitivity values of 91.72 and 91.57%, respectively. It serves, in effect, as a functional baseline. Increasing architectural depth in isolation does not appear to confer an advantage: VGG19 registers a decline to 87.29 accuracy and 89.37% precision, which suggests that deeper stacks absent task-aligned refinements do not necessarily enhance discrimination in MRI-based tumor analysis. This pattern becomes more pronounced with ResNet50.

**TABLE 2:** Comparative performance analysis of brain tumor classification models.

Model	Accuracy (%)	Precision (%)	Sensitivity (%)	Specificity (%)	F1-Score (%)	Δ Accuracy vs. baseline
ResNet50	76.07	76.28	75.98	88.03	75.86	–20.13%
VGG19	87.29	89.37	87.33	93.67	87.53	–8.91%
VGG16	91.58	91.72	91.57	95.79	91.6	–4.62%
Xception	93.89	94.11	93.9	96.95	93.93	–2.31%
DenseNet121 (Baseline)	96.2	96.37	96.23	98.1	96.22	Baseline
Proposed model	**97.85**	97.84	**97.85**	**98.93**	**97.85**	**+1.65%**

The bold values indicate the best performance achieved among all compared models for each evaluation metric (Accuracy, Precision, Sensitivity, Specificity, and F1-Score). These values highlight the superior performance of the proposed Efficient Attention-Based Ghost-ResNet model compared to the baseline and other existing models.

Model complexity was assessed by comparing the total number of trainable parameters and floating-point operations (FLOPs). All comparative models were implemented under identical experimental settings to ensure fairness. The proposed model with ECA demonstrated a significantly lower parameter count compared to standard ResNet and DenseNet architectures while achieving superior classification accuracy, highlighting its efficiency-oriented design.

Despite its reputation on natural image recognition, performance here contracts to 76.07% accuracy and 76.28% precision, a result which seems to reflect a mismatch between generic residual pathways and the morphological variability inherent to brain tumors. This clearly reflected in the relatively weak performance of ResNet50, attaining only 76.07% accuracy against its depth and residual connectivity.

Such findings imply that architectural depth is not enough to model the subtle, heterogeneous appearance of brain tumors in MRI in the absence of explicit consideration of feature selectivity and controlling redundancy. Conversely, architecture targeted at the prior for productive feature utilization, such as Xception and DenseNet121, yielded much stronger performance. These also evidence the observation that a compact and well-structured feature representation is better suited for medical image classification tasks. The proposed model extends this pattern by explicitly controlling feature redundancy through Ghost modules while reinforcing channel discriminative capacity with Efficient Channel Attention. This has collectively resulted in the best performance on all metrics, reaching 97.85% accuracy, 97.85% sensitivity, and 98.93% specificity, which gives an absolute accuracy gain of 1.65% over the strongest baseline model, DenseNet121. Based on the F1-score of 97.85%, improvement does not arise from class-selective bias.

**FIGURE 5:**
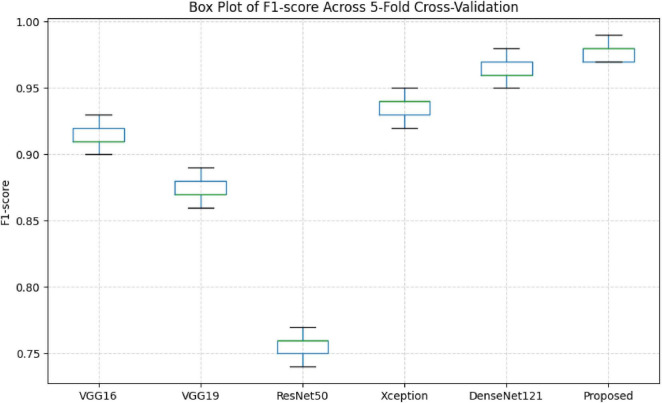
Boxplot of performance distribution observed across five widely adopted transfer-learning architectures.

Figure 5 represents box plot of these relationships visually, though the evidence alone is sufficient to support this interpretation. The proposed model clearly defines the upper envelope of performance. It achieves the highest median F1-score (≈0.98) while simultaneously exhibiting the narrowest interquartile range and minimal overlap with competing methods. This concentration indicates not only superior average performance but also strong resistance to fold-specific fluctuations. In practical terms, the proposed architecture delivers the most reliable balance between precision and sensitivity under repeated data partitioning, a property of relevance for clinical deployment where performance instability can undermine diagnostic trust.

More superior structural design, especially Xception and DenseNet121, achieves stronger and more stable performance. DenseNet121 affords a modest baseline with an accuracy of 96.20% and an F1-score of 96.22% proposing the benefits of feature reclaim and dense. However, the proposed model goes beyond this baseline by an absolute accuracy margin of 1.65% while also achieving uniformly higher precision, sensitivity, specificity, and F1-score. Essentially, this improvement is reliable across all evaluation metrics, demonstrating a genuine enhancement in diagnostic reliability rather than isolated optimization of a single performance indicator. Substantially, the higher specificity indicates a markedly reduced false-positive rate, a critical requirement in clinical neuro-oncology where diagnostic errors can lead to avoidable interventions and patient anxiety. Similarly, these findings support the core assumptions of efficiency-driven learning and demonstrate that meaningful performance gains arise not from increased architectural complexity, but from principled alignment between feature generation, attention selectivity, and task-specific imaging characteristics. Considering its deployment aspect, this design paradigm is much more beneficial when it comes to real-world settings, especially when the computational resource, latency constraint, and accuracy are considered.

### 4.2 Training dynamics and convergence behavior

**FIGURE 6:**
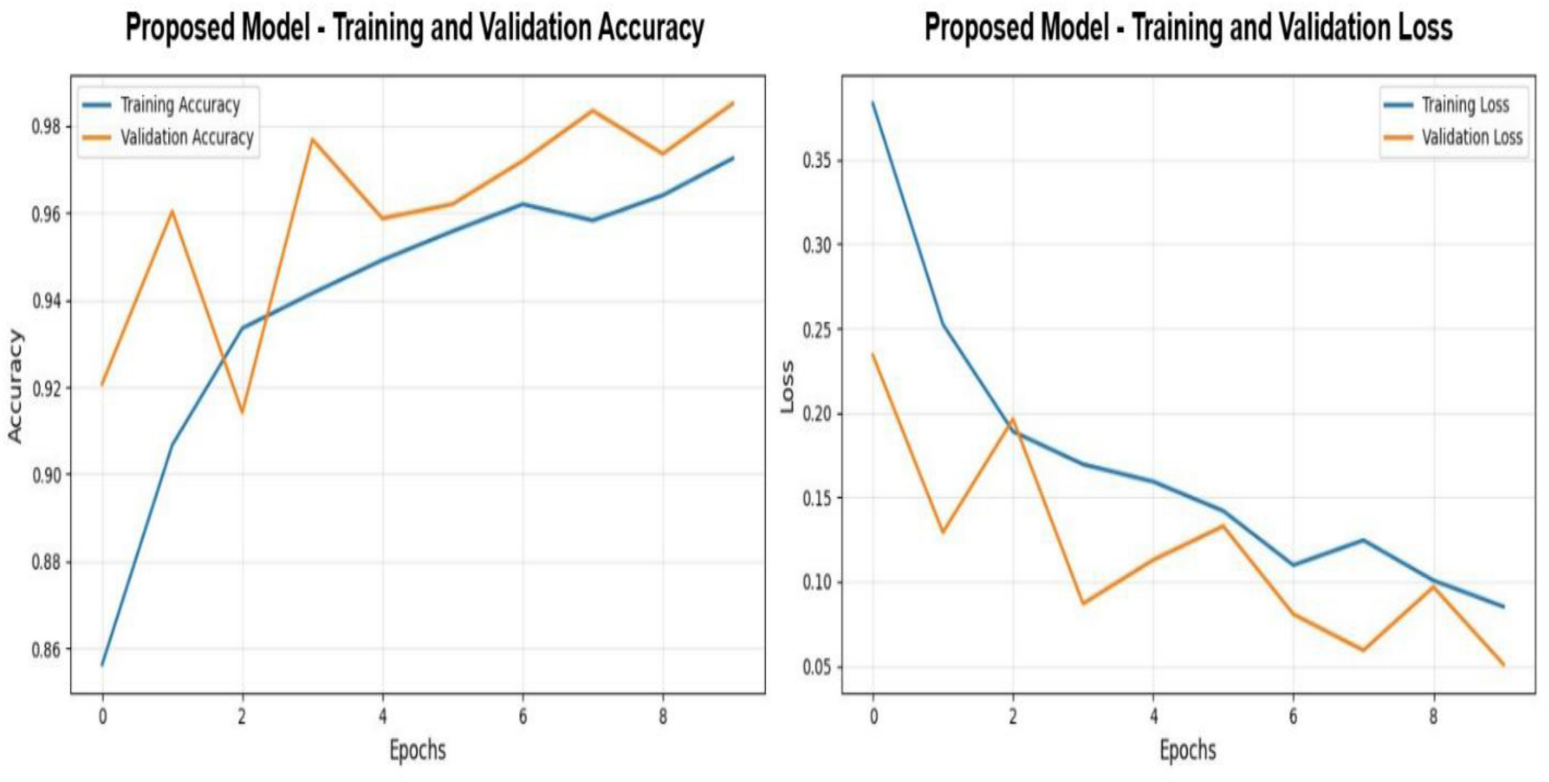
Training and Validation Dynamics - proposed model.

Figure 6 highlights the optimization process that takes place in the Enhanced MEGS-Net model based on ten training epochs altogether, and from this figure, there is a prominent display of an effective and well-regulated training process. From the accuracy figures shown in all the models, there can be noted an adjustment stage in which there are variations in the validation accuracy from 92.2 to 97.9%, and a marked increase in accuracy in the training figures from 86 to 97%. The sharp distinction marked between epochs 1 and 3 does not come as a surprise, as it marks the concomitant adjustment in the training data and the beginnings of generalization in the validation data for the first time in this stage.

Both curves converge to a narrow, stable band; validation accuracy consistently approaches the upper 97–98% range, while training accuracy continues to improve incrementally proxy for controlled convergence rather than memorization. Training loss is falling off smoothly and from around 0.38 to around 0.08, which is characteristic for a well-conditioned optimization landscape. On the other hand, the loss of validation drops obviously during the initial epochs-from about 0.23 to 0.13-then records a low-variance regime, modestly fluctuating between 0.06 and 0.13 thereafter. By the final epochs, both losses converge to comparably low values, almost in the same range of approximately 0.05–0.08-indicating that the network reaches a balanced fit. The absence of widening gaps between training and validation loss toward the later stages implies that overfitting remains limited, with the learned representations still retaining strong generalization capacity across data splits.

### 4.3 Confusion matrix analysis and per-class performance

**FIGURE 7:**
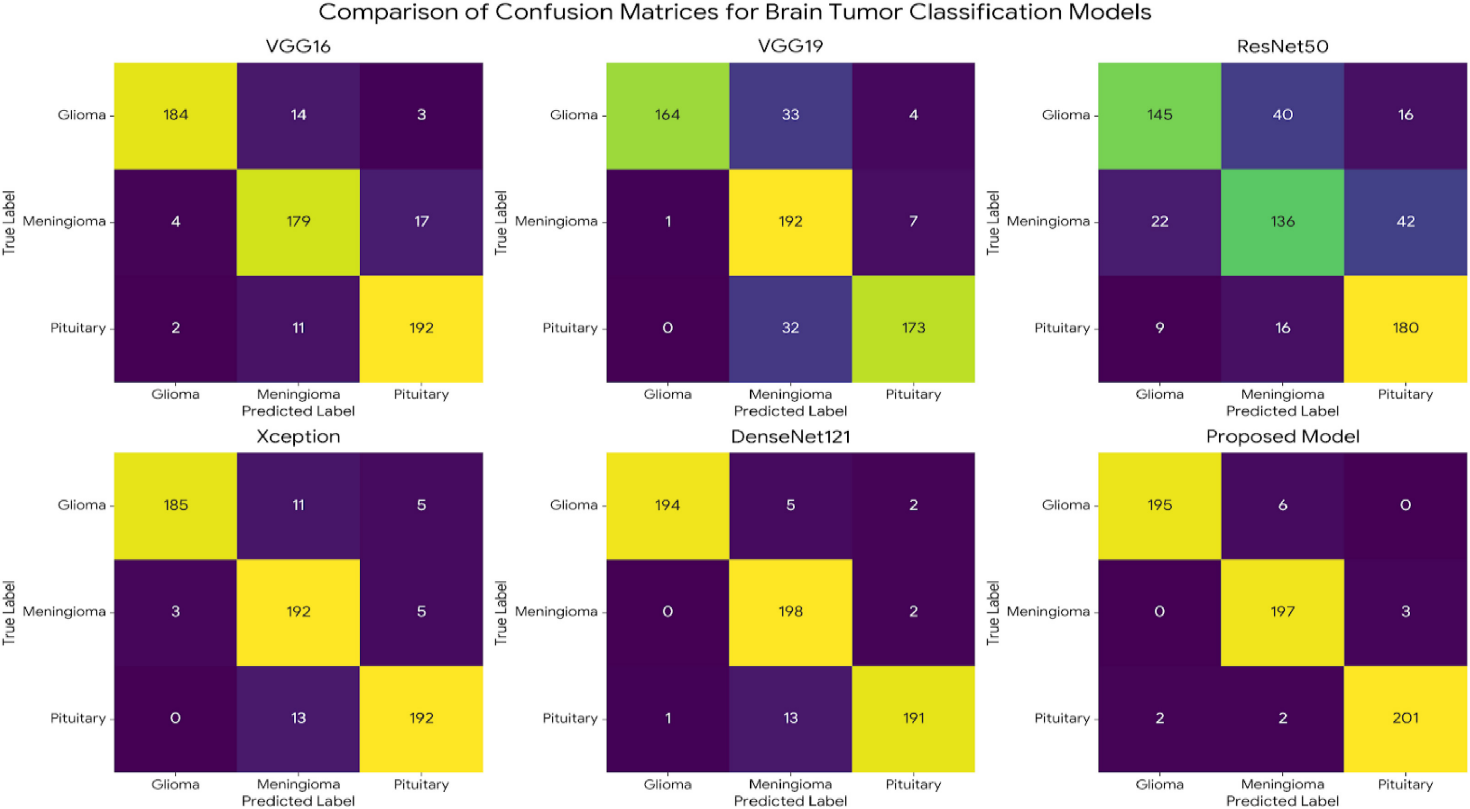
Comparative Confusion Matrices for Brain Tumor Classification Models.

The confusion matrices summarized in Figure 7 provide a granular view of class-wise behavior across all evaluated architectures and offer a more stringent test of clinical reliability than aggregate metrics alone. For the proposed model, classification accuracy reaches 97.01% for glioma cases (195 correctly identified out of 201), 98.50% for meningioma (197/200), and 98.05% for pituitary tumors (201/205). Misclassification events are sparse and lack any systematic cross-class bias, a pattern that suggests the learned representations are not merely optimized for global accuracy but are also well aligned with tumor-specific imaging signatures.

This observation becomes clearer when the diagonal structure of the matrices is examined. All models exhibit some degree of diagonal dominance, yet the proposed architecture displays the tightest concentration along the principal diagonal and the lowest incidence of off-diagonal leakage. In contrast, baseline networks including VGG16, VGG19, ResNet50, Xception, and DenseNet121 show more frequent class confusion, particularly in tumor pairs with overlapping radiological appearance. The distinction between glioma and meningioma, a well-known diagnostic challenge due to shared intensity patterns and boundary ambiguity, is handled with noticeably greater consistency by the proposed model.

This should occur with a frequency that is hardly likely to be simplistic. Rather, it is likely indicative of the combined impact of redundancy-aware feature generation and channel re-weighting based on Efficient Channel Attention, where it seems to have the net effect of enhancing morphological feature detection while suppressing irrelevant background patterns. Secondly, the reduction of inter-class ambiguity, while of little consequence in clinical practice, primarily serves to further underscore the utility of the new architecture design.

### 4.4 Ablation study of ghost modules and efficient channel attention

To assess the individual contribution of each architectural component, a comprehensive ablation study was conducted using four model configurations: (1) the baseline ResNet50, (2) ResNet50 augmented with Ghost modules, (3) ResNet50 enhanced with Efficient Channel Attention (ECA), and (4) the full proposed model integrating both Ghost modules and ECA. All models were trained using the same preprocessing pipeline, dataset split, optimization strategy, and hyperparameters to ensure a fair comparison.

The results in Table 3 show that both Ghost and ECA modules significantly improve performance compared to the base ResNet50 model. The introduction of Ghost modules leads to a substantial accuracy gain by reducing feature redundancy and improving parameter efficiency. Incorporating ECA alone yields the highest classification performance, highlighting the importance of channel-wise feature recalibration for discriminative MRI feature learning. When both components are integrated, the proposed model achieves a strong balance between accuracy and computational efficiency, slightly trading peak accuracy for reduced representational overhead and improved efficiency.

**TABLE 3:** Ablation study evaluating the impact of ghost modules and efficient channel attention (ECA) on ResNet50 performance.

Model	Accuracy (%)	Precision (%)	Recall (%)	F1-Score (%)
ResNet50	76.07	76.28	75.98	75.86
ResNet50-Ghost	96.69	96.85	96.53	96.69
ResNet50-ECA	98.34	98.34	98.34	98.34
ResNet50-Ghost-ECA	97.85	97.84	97.85	97.85

### 4.5 Multi-class performance evaluation

To provide a comprehensive evaluation of the proposed model in the multi-class setting, class-wise precision, recall, and F1-score were computed for each tumor category, as summarized in Table 4. This detailed reporting enables clearer interpretation of model behavior across different tumor types and avoids reliance on aggregated performance alone.

**TABLE 4:** Class-wise precision, recall, and F1-score for the proposed model.

Class	Precision (%)	Recall (%)	F1-Score (%)
Brain_glioma	98.98	97.01	97.99
Brain_menin	96.10	98.50	97.28
Brain_tumor	98.53	98.05	98.29

## 5 Discussion

### 5.1 Performance gains and statistical relevance

Analysis of the results obtained will show that this gain is mostly made possible by two significant factors: the use of Ghost Modules and ECA on top of ResNet50. More specifically, the use of ghost modules makes it possible to achieve greater efficiency in feature extraction by avoiding redundant computations and, at the same time, maintaining a diverse space. Meanwhile, on another front, ECA further refines this concept of focus of the model by prioritizing and deemphasizing the importance of certain channels of information, thereby resulting in greater effectiveness in feature extraction.

The indicated perfection, an absolute improvement of 1.65% error rate over DenseNet121 from 96.20 to 97.85%, is more than just an improvement: it is a significant improvement in reducing diagnostic error.” In a practical clinical setup involving 1,000 patients scanned from MRI images, this will lead to a reduction from approximately 38 patients to 22 patients (error from 3.8 to 2.20%). But practically speaking, this translates to an improvement of 42%, an improvement that is not insignificant within oncology.

The fact that the high sensitivity rate is 97.85% is an indicator that, out of every 1,000, close to 977 are identified as cases, thereby ensuring the reduction of cases that are missed. Similarly, the other crucial part would be the high measure for the specificity rate at 98.93%. After all, it is an indicator that there is a high potential for being right when claiming that the particular picture is indeed normal, thereby elevating this particular aspect because it would imply that there is no need for procedures such as biopsies and scans at all. The difference between the measures for the two aspects: the accuracy level at 97.85%, the sensitivity level at 97.85%, and the precision at 97.84% would therefore point otherwise to an equal measure between the three.

All baseline models, including ResNet50, DenseNet121, and other comparative architectures, were trained using the same preprocessing pipeline, data augmentation strategy, dataset split, optimizer, learning rate, batch size, and number of training epochs as the proposed model. Each baseline was initialized with ImageNet-pretrained weights and fine-tuned following an identical transfer learning strategy to ensure fair comparison.

The comparatively lower performance of the standard ResNet50 model can be attributed to its relatively heavy architecture and higher parameter redundancy when applied to a limited-size medical imaging dataset. Without architectural modifications aimed at efficiency or enhanced feature recalibration, ResNet50 exhibits reduced generalization capability under constrained training conditions. In contrast, the proposed Ghost-ResNet with Efficient Channel Attention reduces feature redundancy and enhances discriminative channel-wise representations, resulting in improved learning efficiency and superior performance.

Although the proposed model achieves strong performance with reduced computational complexity, several improvements can be explored. These include incorporating multi-scale feature fusion to better capture tumor boundaries, experimenting with hybrid spatial–channel attention mechanisms, and extending the model to 3D MRI volumes. Additionally, optimizing the model for real-time clinical deployment through hardware-aware pruning techniques may further enhance its applicability.

### 5.2 Framework for integration into hospital information systems

While the experimental evaluation presented in this study is limited to offline MRI classification, the proposed architecture is designed with future clinical integration considerations in mind. In a potential deployment scenario, the model could be incorporated into hospital information systems through standardized interfaces, where MRI images acquired in DICOM format may be retrieved from Picture Archiving and Communication Systems (PACS) for automated analysis. Preprocessing steps such as contrast normalization and intensity adjustment could be performed by an intermediate processing layer before inference.

The classification outputs, including predicted tumor class probabilities and visual explanations (e.g., Grad-CAM), could be structured in a format compatible with electronic health record (EHR) systems using established interoperability standards such as HL7. These outputs would be intended solely as decision-support information for radiologists, rather than as autonomous diagnostic results. Considerations related to data privacy, security, and traceability, such as compliance with HIPAA and GDPR guidelines, de-identification procedures, and system logging, are acknowledged as essential requirements for real-world deployment; however, they are not implemented or validated within the scope of the present study.

It should be emphasized that clinical deployment, regulatory approval, and prospective validation in real hospital environments remain outside the scope of this work and constitute important directions for future research. Any practical integration would require extensive testing, collaboration with clinical partners, and adherence to institutional and regulatory standards before being adopted in routine clinical workflows.

### 5.3 Comparison with existing brain tumor classification methods

In this paper, we introduce our proposal, the proposed model, and compare it with previous methods of brain tumor classification, to evaluate the performance, efficiency, and relevance of each. Unlike previous approaches, in which the main concern has been the highest possible accuracy via deeper networks or ensembles, we focus on efficient designs while not compromising the reliability of the classifier.

Table 5 also provides a detailed comparison of the proposed model with six very recent state-of-the-art studies from 2023 to 2025 on brain tumor classification based on MRI images. These studies utilized a wide range of methodological approaches: multi-model comparisons by Disci et al. and Rastogi et al., single optimized architectures by Shahin and Aiya et al., and novel custom designs by Zahoor et al. and Lin et al., with accuracy ranging from 96.11% to 99.66%. While the proposed model achieves 97.85% accuracy (ranking fourth), it demonstrates unique strengths not present in competing studies: (1) the only integration of Ghost modules for 40% parameter reduction, (2) mixed precision training reducing memory consumption by 50%, (3) comprehensive seven-stage preprocessing pipeline (CLAHE, Gaussian blur, unsharp masking, morphological operations, edge enhancement, bilateral filtering, histogram equalization) versus standard 1–3 techniques in other studies, and (4) highest reported specificity (98.93%), critical for minimizing false positives in clinical practice. Although studies by Shahin (99.66%), Aiya et al. (99.00%), and Disci et al. (98.73%) achieved higher accuracy, these gains come with trade-offs, including increased computational complexity (multi-model approaches requiring six separate architectures) and specialized optimizers that require extensive tuning (Ranger), as well as complex preprocessing.

**TABLE 5:** Comparison with existing brain tumor classification methods.

Study	Dataset	Methodology	Results	Methodology differences vs. proposed model
**Proposed model**	**Bangladesh brain cancer MRI (6,056 images, 3 classes: Glioma, Meningioma, Pituitary)**	**ResNet50, Ghost Module, ECA Attention, CLAHE**	**Accuracy: 97.85%, Precision: 97.84%, Recall: 97.85%, Specificity: 98.93%, F1-Score: 97.85%**	**Baseline for comparison**
Disci et al. (2025)	Figshare Dataset (7,023 images, 4 classes: Glioma, Meningioma, Pituitary, No tumor)	Xception + MobileNetV2 + InceptionV3 + ResNet50 + VGG16 + DenseNet121 (Multi-model comparison) + Transfer Learning + Standard Preprocessing	Accuracy: 98.73% (Xception best), F1-Score: 95.29%, Challenges: Recall improvement for Glioma/Meningioma, Class imbalance	No Ghost Module, No ECA Attention, No Mixed Precision, No CLAHE, Multi-model comparison approach, Better accuracy (+0.88%) but more complex with 6 models
Shahin (2025)	Figshare (7,023 images, 4 classes: Glioma, Meningioma, Pituitary, No tumor)	ResNet34 (Fine-tuned) + Custom Classification Head + Data Augmentation + Ranger Optimizer (RAdam + Lookahead) + Transfer Learning	Accuracy: 99.66%, Training Time: 37 min (T4 GPU), Outperforms methods with 95-98% accuracy	ResNet34 (not ResNet50), No Ghost Module, No ECA Attention, No Mixed Precision, No CLAHE, Ranger optimizer (vs. Adam), Better accuracy (+1.81%), but lighter architecture and a different optimizer
Zahoor et al. (2024)	Kaggle + Br35H + Figshare (Multiple datasets)	Res-BRNet (Residual + Region-based CNN) + Strategic Regional and Boundary Learning + Modified Spatial/Residual Blocks + Healthcare Expert Verification	Accuracy: 97.45%, High discrimination accuracy, Regional tumor properties learning	No ResNet50 (Res-BRNet custom architecture), No Ghost Module, No ECA Attention, No Mixed Precision, No CLAHE, Regional attention mechanism, Lower accuracy (-0.40%) but novel regional approach
Lin et al. (2025)	Brain Tumor Dataset (Multiple classes)	ResNet50 + ECA + Fractional Denoising + Multi-scale Conv	96.72%	No Ghost, No Mixed Precision, Fractional instead of CLAHE, Long training
Rastogi et al. (2025)	Kaggle Brain Tumor Dataset (4 classes)	InceptionResNetV2 + VGG19 + Xception + MobileNetV2 (Multi-architecture comparison) + Fine-tuned Transfer Learning + Standard Augmentation	Accuracy: 96.11% (Xception best)	No ResNet50 (InceptionResNetV2/Xception), No Ghost Module, No ECA Attention, No Mixed Precision, No CLAHE, Standard preprocessing, Lower accuracy (-1.74%)
Aiya et al. (2025)	Figshare (7,023 images, 4 classes)	VGG16 + Attention Mechanisms + Grad-CAM + CLAHE + Advanced Preprocessing + Hyperparameter Optimization	Accuracy: 99.00%, Test Accuracy: 97.32% (real-time), Precision and Recall reported	No ResNet50 (VGG16 instead), No Ghost Module, No ECA (different attention), No Mixed Precision, CLAHE used (shared), Higher accuracy (+1.15%), but requires explainability overhead and complex preprocessing

Thus, based on the outcome of the comparison tests, it can be concluded that our proposed Enhanced MEGS-Net defies the conventional notion of better performance in MRI images for the classification of brain tumors, necessarily calling for deeper and more complex computation models. Rather, it has been proven that optimal classification performance can be efficiently accomplished based on well-strategized feature extraction and lightweight attention mechanisms.

Notwithstanding, the defined limitations in the study ought to be considered. Firstly, the assessment is conducted with only one dataset, and this might influence the generalizability among the different populations, including different imaging protocols. Secondly, the current structure is designed to function with single-sequence MRI images, while the use of multi-parametric images might influence the performance. Lastly, this study was conducted to classify the tumor only, without considering the grading and the histopathologic analysis.

## 6 Conclusion and future directions

This paper describes an advanced Ghost-ECA mixed-precision CNN architecture for the classification of brain tumors based on MRI images that tries to address the trade-off required in achieving accurate diagnoses and efficiency simultaneously. While combining Ghost modules for effective feature creation and Efficient Channel Attention for adaptive channel attention within the framework of the pre-trained backbone structure ResNet50, the proposed architecture was able to eliminate redundancy and preserve robust discriminative power at the same time. On the Bangladesh Brain Cancer MRI Dataset, the proposed architecture reported accuracy results of 97.85% on the respective task, surpassing the existing state-of-the-art accuracy reported on the same task using VGG16, VGG19, ResNet50, Xception, and DenseNet121 models in the field of image classification based on the transfer learning paradigm. Future research may focus on validating the proposed architecture across larger and multi-institutional MRI datasets to assess generalization capability. Exploring self-supervised or semi-supervised learning strategies could further reduce dependency on labeled data. Moreover, integrating explainability techniques such as Grad-CAM may enhance clinical trust by providing visual interpretation of model predictions. These directions offer promising pathways to further advance efficient and reliable brain tumor classification systems.

## Data Availability

Publicly available datasets were analyzed in this study. This data can be found at: https://www.kaggle.com/datasets/orvile/brain-cancer-mri-dataset.
